# Clearing a path for light through non-Hermitian media

**DOI:** 10.1515/nanoph-2024-0140

**Published:** 2024-08-07

**Authors:** Utsav D. Dave, Gaurang R. Bhatt, Janderson R. Rodrigues, Ipshita Datta, Michal Lipson

**Affiliations:** Columbia Nano Initiative, 5798 Columbia University, New York, NY, USA; Department of Electrical Engineering & Columbia Nano Initiative, 5798 Columbia University, New York, NY, USA; Ginzton Laboratory, Stanford University, Stanford, CA, USA

**Keywords:** non-Hermitian, lossy media, metal clad waveguides, thermo-optic

## Abstract

The performance of all active photonic devices today is greatly limited by loss. Here, we show that one can engineer a low loss path in a metal-clad lossy multi-mode waveguide while simultaneously achieving high-performance active photonic devices. We leverage non-Hermitian systems operating beyond the exceptional point to enable the redistribution of losses in a multi-mode photonic waveguide. Consequently, our multi-mode waveguide offers low propagation losses for fundamental mode while other higher order modes experience prohibitively high losses. Furthermore, we show an application of this non-Hermitian waveguide platform in designing power-efficient thermo-optic phase shifters with significantly faster response times than conventional silicon-based thermo-optic phase shifters. Our device achieves a propagation loss of less than 0.02 dB μm^−1^ for our non-Hermitian waveguide-based phase shifters with high performance efficiency of *P*_*π*_ ⋅ *τ* = 19.1 mW μs. In addition, our phase shifters have significantly faster response time (rise/fall time), *τ* ≈ 1.4 μs, compared to traditional silicon based thermo-optic phase shifters.

## 1 Introduction

Active photonic devices suffer from a trade-off between performance and propagation losses due to the placement of loss inducing media in proximity to the waveguide. These lossy materials include metals, heavily doped regions, tight waveguide bends, and other leaky structures that are otherwise essential to achieve the required optical performance. This fundamental trade-off between performance efficiency and insertion loss is integral to the operation of active photonic devices such as thermo-optic phase shifters and electro-optic modulators [1], [2], [3]. For example, the performance of a thermo-optic phase shifter is characterized by the product of power required for phase change (*P*_*π*_) and temporal response of such a phase shifter (*τ*). One can improve the performance of thermo-optic devices (*τ* < 1 μs) by placing the heating elements (usually a metal) in proximity to the waveguide. However, this comes at an expense of unacceptably high propagation losses of up to 10 dB [2]. To circumvent the propagation losses, conventional photonic device design places metals on the cladding several microns away, consequently increasing operational power requirements and response times. This ultimately leads to lower performance efficiency (i.e. increase in *P*_*π*_ ⋅ *τ*). A similar performance trade-off exists for electro-optic modulators, where the performance (modulation depth and speed) is impacted by the relative placement of metal electrodes with respect to the waveguide when optimizing the design for lower propagation losses [4], [5], [6], [7], [8], [9], [10], [11], [12].

## 2 Non-Hermitian waveguides

**Figure 1:**
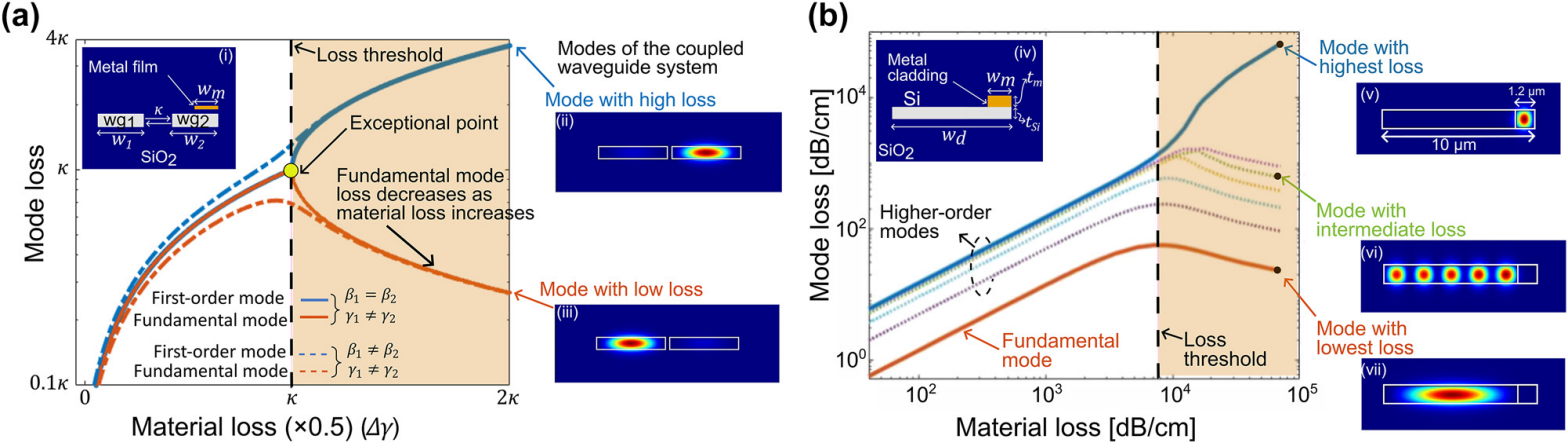
A system of non-Hermitian photonic waveguides. (a) Evolution of mode loss for the first symmetric (solid red line) and asymmetric (solid blue line) mode of a coupled waveguide system as a function of waveguide’s intrinsic loss difference (i.e. Δ*γ* = (*γ*_1_ − *γ*_2_)/2) created by adding material loss. Here the non-Hermitian system consists of two identical coupled waveguides, wg_1_ and wg_2_, having widths such that *w*_1_ + *w*_2_ = *w*_*d*_ (*w*_1_ = *w*_2_, *β*_1_ = *β*_2_, *γ*_1_ = 0, *γ*_2_ > 0), and coupling rate *κ*. The dotted lines presents the case for non-identical coupled waveguides with *β*_1_ ≠ *β*_2_ (*w*_1_ ≠ *w*_2_). The insets ii and iii, present the high and low loss modes of a coupled silicon waveguide system. (b) Propagation losses of various modes of a wide multi-mode silicon waveguide as a function of intrinsic material loss. Unlike plot (a), in this plot the coupled waveguide system with unequal losses is created by using a wide waveguide with partial metal clad region (see inset iv). The simulation results shown here are performed at a single wavelength *λ*_0_ = 1.6 μm. Similar results can be generated for any operating wavelength.

Here, we show an approach that overcomes this trade-off and permits the placement of metal in proximity to waveguides without introducing additional losses. We achieve this by designing multi-mode waveguides and redistributing system losses such that the loss of fundamental mode is shifted to the higher order modes, while keeping the overall loss of the system constant. This helps us engineer a low-loss light path for the fundamental mode through a lossy media. Similar loss redistribution mechanisms have been theoretically analyzed and demonstrated in coupled waveguide systems for achieving optical transparency, for single mode lasing from ring-resonators via gain-loss balancing, and other non-integrated photonic systems, but not in integrated multi-mode waveguides [13], [14], [15], [16], [17], [18], [19]. One can explore this idea of loss redistribution by looking at the evolution of the mode loss in a coupled waveguide system, with distributed intrinsic losses between two coupled waveguides, as shown in Figure 1a. In this, we show the evolution of the symmetric and asymmetric mode (red and blue line respectively) of a coupled waveguide system as a function of intrinsic waveguide loss. The system consists of two identical waveguides (*β*_1_ = *β*_2_) of widths *w*_1_ and *w*_2_ (*w*_1_ + *w*_2_ = *w*_*d*_) having certain coupling rate *κ*, but distinct losses *γ*_1_ and *γ*_2_  (*γ*_1_ ≠ *γ*_2_), respectively. We choose *γ*_1_ = 0 for waveguide wg_1_, and sweep the intrinsic loss of the second waveguide wg_2_ such that *γ*_2_ > 0 by assuming an arbitrary metallic film of width *w*_*m*_ placed on top as cladding. Here, *γ*_2_ depends on the optical properties of the metal film and its proximity to the waveguide. We consider two distinct cases, where – (i) the waveguides are identical (solid lines in Figure 1a), and (ii) the waveguides are non-identical (*w*_1_ ≠ *w*_2_, *β*_1_ ≠ *β*_2_, and, Δ*β* = (*β*_1_ − *β*_2_)/2) shown by broken lines in Figure 1a. One can see that the dependence of the mode loss on the differential loss (Δ*γ* = (*γ*_1_ − *γ*_2_)/2) between the waveguides, exhibits the typical pattern of a coupled non-Hermitian system (see Supplementary Section 1). When Δ*γ* = *κ*, the two mode losses converge resulting in an exceptional point (EP), represented by a yellow circle on the plot in 1a. Beyond this point (threshold loss), an increase in Δ*γ* (Δ*γ* ≫ *κ*) leads to a decrease in mode loss for the asymmetric mode and an increase in loss for symmetric mode. By designing the waveguides with appropriate difference in propagation constant, the threshold can be engineered for a coupled system. For this example, we have considered conventional silicon waveguides with identical widths *w* = 450 nm and heights *h* = 220 nm. In order to introduce realistic waveguide losses and change in propagation constant, we consider aluminum as cladding material placed partially covering the wg_2_ (*w*_*m*_ = 200 nm). This slight asymmetry in the design leads to non-identical waveguides (Δ*β* ≠ 0) with unequal losses (Δ*γ* > 0). For computational results presented in Figure 1a, the plot axes are normalized with respect to the coupling rate *κ*.

**Figure 2:**
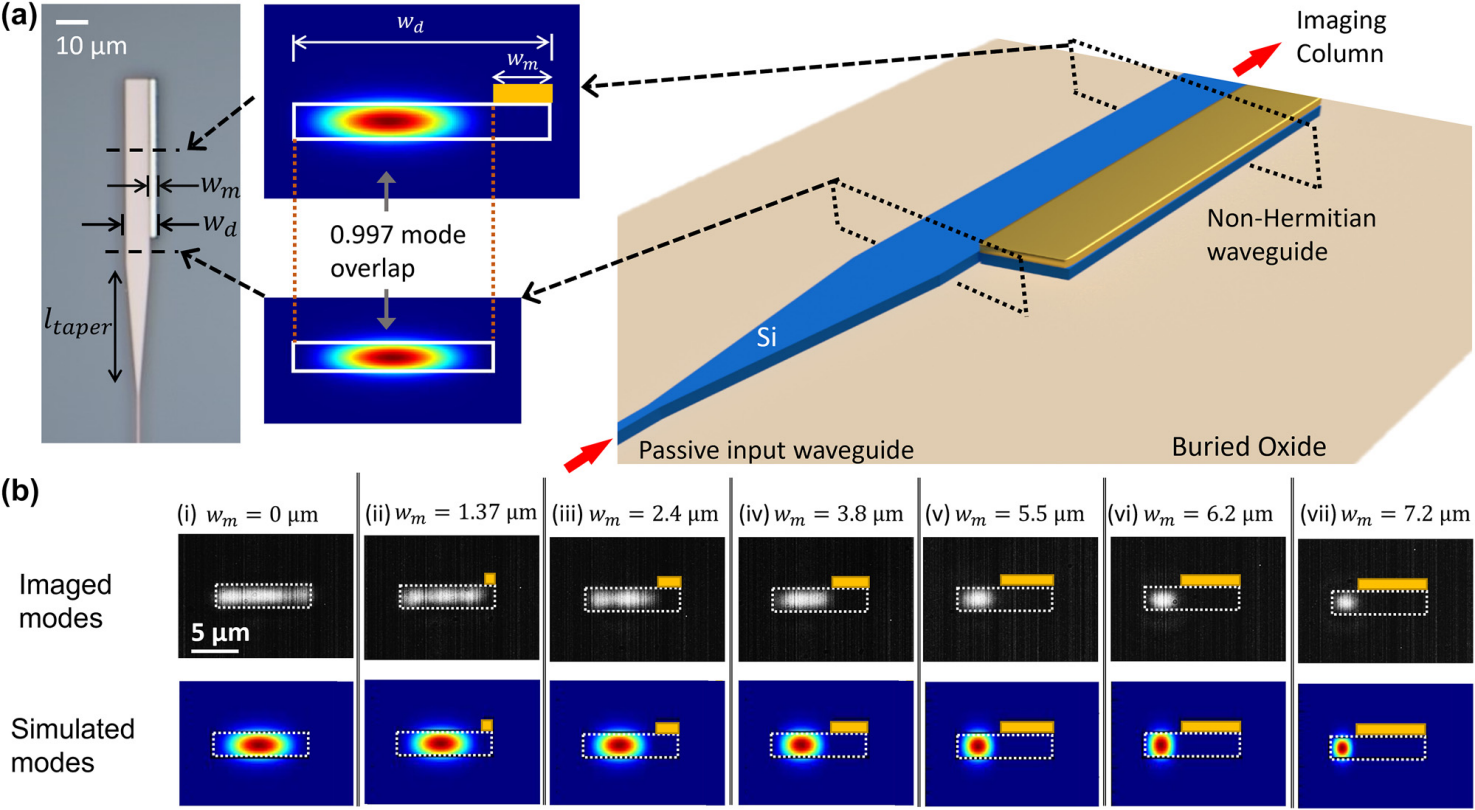
A multi-mode silicon based non-Hermitian waveguide with lossless fundamental mode. (a) (left panel) Microscope image of different components of the device. The device consists of a single mode silicon waveguide (*w*_SM_ = 450 nm), waveguide taper (*l*_taper_ = 30 μm) from single mode width to the unclad region of the multi-mode waveguide, and the non-Hermitian waveguide with partial (*w*_*m*_) aluminum cladding. The taper is used to excite the fundamental low loss mode of the non-Hermitian waveguide. (middle panel) Simulated fundamental mode of the unclad multi-mode waveguide, and the low loss mode of the non-Hermitian waveguide, along with their mode overlap (Γ_overlap_ = 0.997). (right panel) Schematic of the device used to image the modes. Our design ensures that there is maximum overlap between the fundamental mode of the unclad multi-mode waveguide and the low-loss mode of the non-Hermitian waveguide. (b) The modes of non-Hermitian waveguides with a silicon waveguide width of *w*_*d*_ = 10 μm, and partial aluminum cladding width (*w*_*m*_) ranging from 0 to 7.2 μm with a thickness of *t*_*m*_ = 120 nm for columns (i) to (vii). With an increasing metal width, the loss of the material remains beyond the threshold, which ensures mode confinement to the low-loss section. Top row panels show the experimentally imaged mode while the corresponding panel in bottom row represents the simulated mode profile of waveguide with equivalent dimensions of *w*_*d*_ and *w*_*m*_. The simulation results shown here are obtained using Lumerical MODE solutions at the wavelength corresponding to the measurement wavelength, *λ*_0_ ≈ 1.6 μm.

We extend the concept of loss redistribution to the multi-mode regime, by designing a coupled non-Hermitian system using a multi-mode silicon waveguide partially covered with a lossy metal film. In this case, the waveguide geometry controls the coupling rate while the material loss in the system is controlled by varying one or more of the geometrical parameters of the metal film covering the waveguide (i.e. coverage width, film thickness and/or distance from waveguide). Figure 1b shows the computed propagation losses of the fundamental and first six of the higher order modes as a function of material loss. One can see the redistribution of the loss for a sufficiently high material loss (shaded region in Figure 1b), where the propagation loss of the low-order modes decreases with an increase in the material loss, while the propagation loss of the higher order mode increases. The fundamental mode ultimately exhibits propagation losses many orders of magnitude lower than the system losses present in part of the non-Hermitian waveguide. In this computation, we have assumed an overall unclad silicon waveguides with a partial cladding of aluminum metal with a width *w*_*m*_ = 1.2 μm. We vary the distance of the partially clad metal film to achieve required material losses in the range of 40 dB cm^−1^ − 7 × 10^4^ dB cm^−1^ for a constant thickness of *t*_*m*_ = 120 nm. We obtain a loss threshold value of approximately ≈7 × 10^3^ dB cm^−1^ for creating modes with distinct losses. Beyond this loss threshold, a low loss mode emerges presenting a propagation loss of ≈22 dB cm^−1^, even when the material loss is ≈7 × 10^4^ dB cm^−1^. In Figure 2b we also present simulated mode profiles of the low and high loss modes in the inset v, vi, vii. One can see from the right inset of Figure 1b that the fundamental mode (also the low loss mode) is solely confined to the waveguide region without any metal covering (low-loss region). In contrast, the higher order modes gradually extend into the region of the waveguide covered by metal, leading to their relatively higher losses. The multi-mode waveguide considered here is of width *w*_*d*_ = 10 μm, height *h* = 220 nm and metal coverage *w*_*m*_ = 1.2 μm. Detailed theory and mathematical formulation of the above description is provided in Supplementary Section  1.

## 3 Results and discussions

We implement the non-Hermitian system described above by fabricating a multi-mode waveguide with partially covered aluminum cladding in SOI platform and demonstrate that the fundamental mode is confined solely to the low-loss region. The fabricated device consists of 10 μm wide silicon waveguides, partially clad with aluminum film (*n*_Al_ = 1.44 + 15.9*j*) of thickness *t*_*m*_ = 120 nm and varying widths ranging from *w*_*m*_ = 0 μm to *w*_*m*_ = 7.2 μm. Here, the width of the metal coverage on the waveguide determines the intrinsic waveguide losses. The light is coupled into the non-metalized section of the non-Hermitian waveguide from the single-mode waveguide using waveguide tapers (*l*_taper_ = 30 μm) as shown in Figure 2a. The taper length is designed to provide slow transition from a single-mode to multi-mode with minimal change in insertion loss and orthogonality. Details on device fabrication are provided in Supplementary Section 2.

We image the edge-emitted optical mode from partially metal-clad waveguides and observe a non-Hermitian, low-loss fundamental mode. In order to image the edge-emitted mode cross-section in the far-field, we use a custom-built optical column comprising of a high magnification objective (Mitutoyo 20×) and an IR camera (Xenics Bobcat 640) at the output of the multi-mode waveguide. Figure 2b shows the experimentally imaged mode cross-section from the partially clad multi-mode silicon waveguide. One can see from the captured mode profiles that the fundamental mode in the multi-mode waveguides with varying metal-clad widths remains confined to the unclad (region without metal) section of the waveguide. Also shown alongside are the corresponding mode profiles obtained via eigen-mode simulations. The imaged modes show good agreement with the corresponding, simulated, waveguide modes with different metal cladding widths, thus confirming the existence and pure excitation of the non-Hermitian fundamental mode. Clearly, the intrinsic losses introduced in the waveguide in the form of partial metal cladding exceed the threshold for this coupled system, ultimately resulting in a low-loss fundamental mode that is confined in the waveguide section without metal cladding. It is worth mentioning here that, there are no exceptional points for systems with asymmetric loss regions but only avoided crossings. In the simulations here, we estimate the losses in the waveguide by computing the fundamental eigen mode of the silicon waveguide with partial Al-cladding on the top and with underlying buried oxide of thickness *t*_BOX_ = 2 μm.

**Figure 3:**
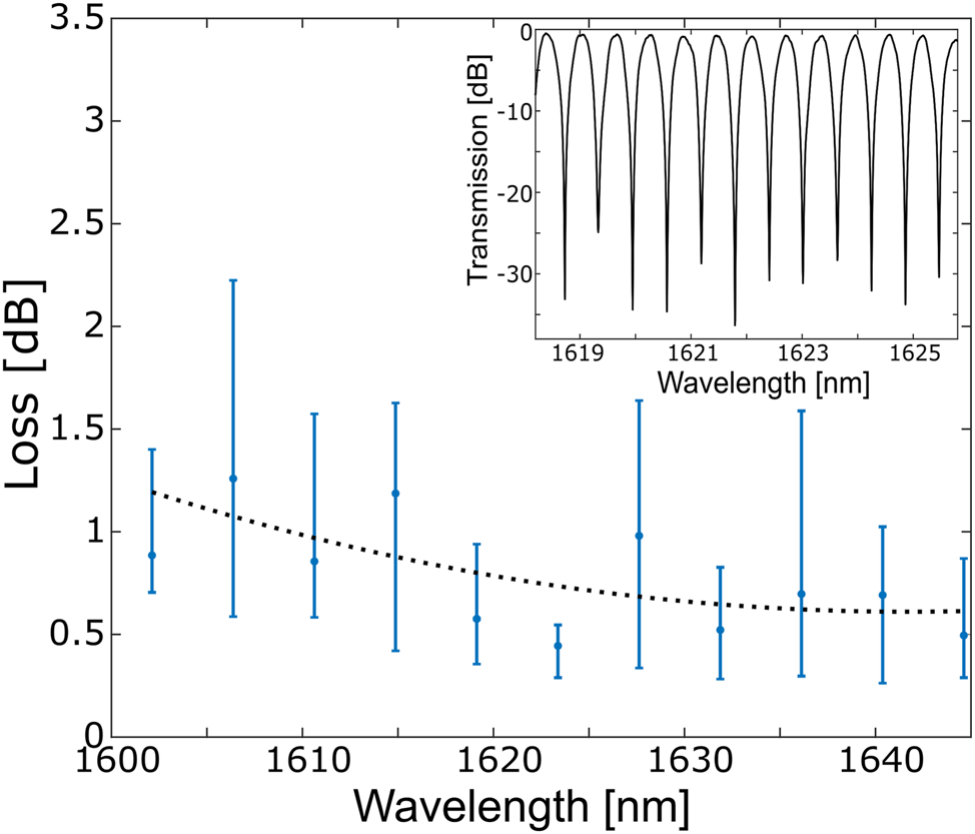
Loss measurements of the fundamental mode in a multi-mode silicon based non-Hermitian waveguide. Measured spectral loss of our non-Hermitian waveguides with waveguide width *w*_*d*_ = 10 μm, aluminum cladding on top for a width *w*_*m*_ = 3.6 μm and a thickness *t*_*m*_ = 120 nm. The loss is extracted from the MZI transmission shown in inset.

We measure a propagation loss of 1.2 ± 0.6 dB for the fundamental mode of a 10 μm wide SOI waveguide with partial metal cladding. For these measurements, we choose a waveguide with a partial metal coverage of *w*_*m*_ = 3.6 μm, placed directly on top of the waveguide, that ensures operation beyond the threshold. In order to measure the propagation loss of the fundamental mode of a non-Hermitian waveguide, we embed our partially metal clad multi-mode silicon waveguide in one of the arms of a length unbalanced Mach–Zehnder interferometer (MZI) (Δ*L* = 1 mm) [20], [21], [22]. The MZI consists of a single mode silicon waveguide with width *w*_*d*_ = 450 nm, and the multi-mode waveguide section in one of the arms runs for a length of *l*_MM_ = 75 μm, with a taper length on each side of ≈150 μm, that ensures seamless transition (and lower coupling losses) from fundamental to multi-mode (and vice versa) while ensuring mode orthogonality. The optical transmission of the composite MZI structure is recorded in the operating wavelength regime from 1.600 to 1.650 μm. Subsequently the insertion losses of the entire device are estimated by fitting the spectral data to the MZI equation (see Supplementary Section 3). Figure 3 shows the extracted spectral loss of our silicon multi-mode waveguide along with recorded MZI transmission in the inset. One can see from the MZI characteristics that our waveguide supports low loss transmission of the fundamental mode, with a total loss propagation loss of ≈1.2 ± 0.6 dB (equivalent to $<0.02\;dB\;{\mathrm{\mu m}}^{-1}$ ), despite having a partial metal cladding. For comparison, an equivalent reference MZI device formed entirely of a single mode waveguide (*w*_*d*_ = 450 nm, air-clad on top) shows propagation losses of 0.008 dB μm^−1^. The transmission measurements performed and discussed above are done on a standard photonic device testing setup. We use a lensed fiber to couple light in/out of the photonic device. The input and output ends consist of inverse tapers for efficient coupling of light between fiber and waveguide [23]. The spectral measurements are recorded by performing a laser wavelength sweep.

**Figure 4:**
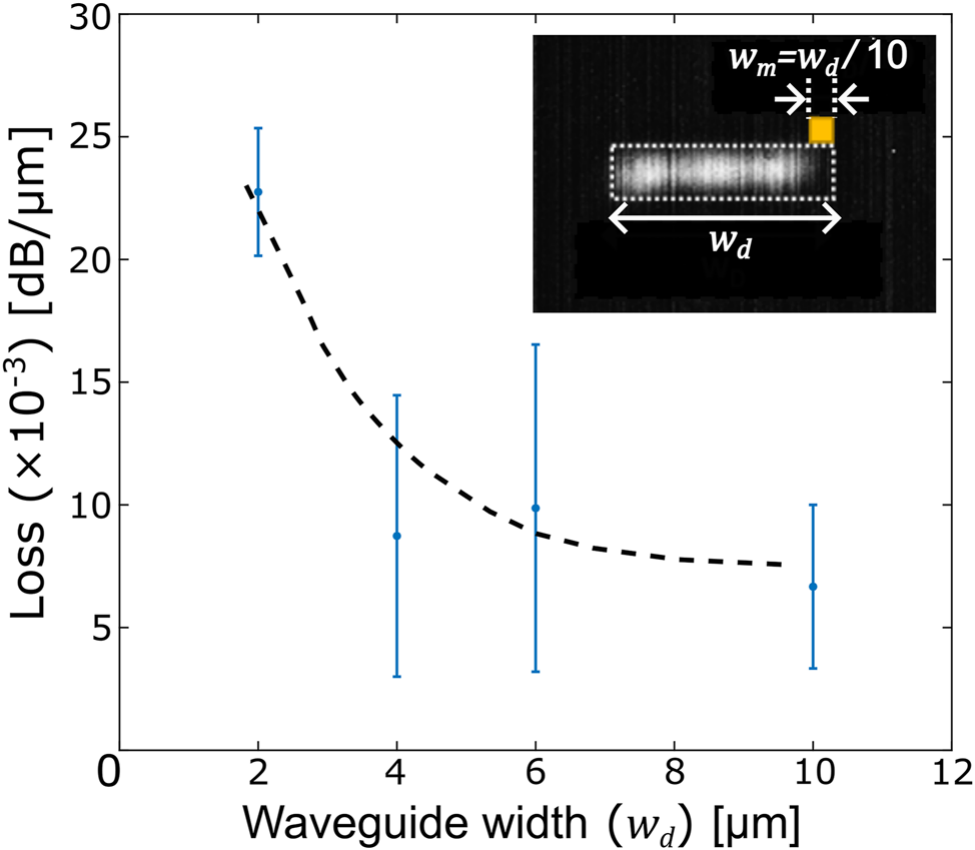
Propagation losses of the fundamental mode in a multi-mode non-Hermitian waveguide of varying widths. Extracted loss at λ_0_ = 1.6 μm wavelength for different widths of the SOI waveguide each with 10 % of its width covered with metal on top. Such an arrangement is chosen to ensure the material loss imparted by the metal remains approximately the same. One can see that as the width of the SOI waveguide increases the mode loss decreases because the threshold for lowering the loss decreases. In all cases however, the mode loss is many orders of magnitude lower than the intrinsic metal loss at this wavelength which is $>500\;dB\;{\mathrm{\mu m}}^{-1}$ .

As an example of non-Hermitian multi-mode waveguide based device, we engineer the waveguide geometry and show a thermo-optic phase shifter that operates with both low-loss as well as high-performance efficiency (lower *P*_*π*_ ⋅ *τ*). The efficiency of thermo-optic phase shifters is improved by reducing the power to achieve *π* phase shift (*P*_*π*_) while simultaneously ensuring a faster response time (*τ*). We utilize our partial aluminum cladding that not only allows us to introduce higher than threshold intrinsic losses in the waveguide but also serves a dual purpose of acting as a heater of the thermo-optic phase shifter. In order to engineer a high-performance device we optimize the placement of cladding metal by having a short distance between the metal and waveguide for faster thermal response time, while simultaneously ensuring low propagation losses for the fundamental mode. We fabricate non-Hermitian multi-mode waveguides with varying widths (*w*_*d*_) and with approximately 10 % of their widths clad with a fixed thickness (*t*_*m*_ ≈ 120 nm) of aluminum (i.e. $w_{m}≅\frac{w_{d}}{10}$ ). Here, the chosen design parameters ensure that the losses introduced by metal coverage are significant enough to obtain performance characteristic as predicted in Figure 1, yet not too high that it will introduce unwanted residual losses to the propagating modes. In order to characterize our devices, we embed our multi-mode waveguides (length *L*_*D*_ = 75 μm) into one arm of a single mode waveguide-based Mach–Zehnder Interferometer and perform interferometric loss measurement, as the one described earlier. The devices used here, as well as all subsequent device results reported in this manuscript possess a single-mode to multi-mode waveguide taper length *l*_taper_ = 150 μm. Figure 3-inset shows the recorded MZI spectrum, while Figure 3 presents the extracted spectral losses. The losses appear lower at longer wavelengths due to stronger mode interaction with the metal cladding, leading to a lower threshold and the existence of low-loss fundamental mode. Figure 4 shows the extracted propagation losses of the fundamental mode for multi-mode waveguide with varying widths at wavelength *λ*_0_ = 1.6 μm. One can see that as the width of the SOI waveguide increases, the loss of the fundamental non-Hermitian mode decreases and it is always orders of magnitude lower than the overall system loss, as predicted in Figure 1b. These propagation losses, even for relatively narrow multi-mode waveguides (*w*_*d*_ = 2 μm), are small enough to enable applications that could use these non-Hermitian waveguides for a wide variety of photonic devices.

**Figure 5:**
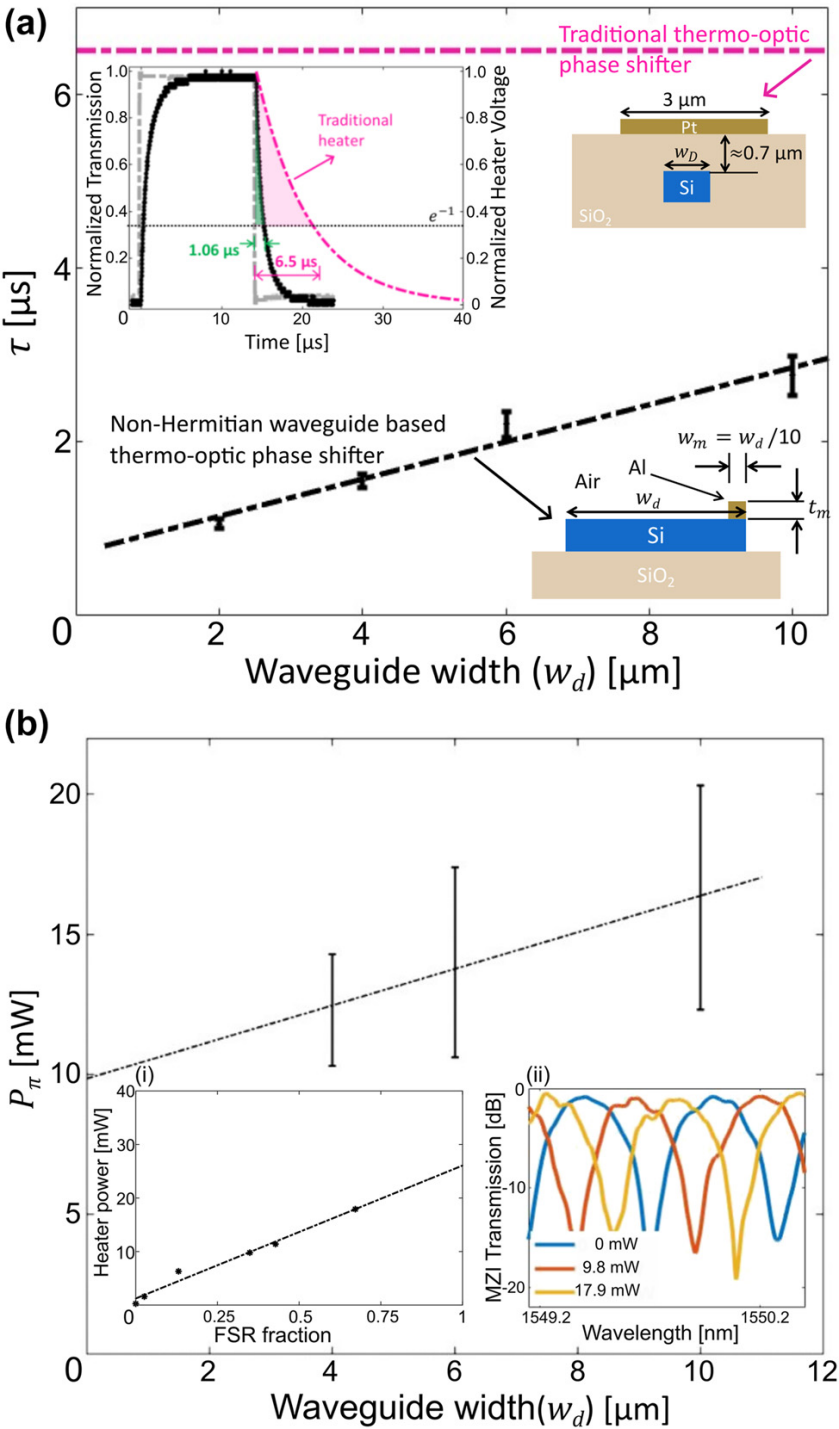
Characteristics of a non-Hermitian waveguide based thermo-optic phase shifter. (a) Measured response time of the non-Hermitian waveguide heaters as a function of the silicon waveguide width. The typical response time of a traditional heater is shown in magenta dashed line for reference. Inset shows measured time response of our thermo-optic phase shifter composed of the non-Hermitian waveguide with width *w*_*d*_ = 2 μm, aluminum cladding width *w*_*m*_ ≈ 270 nm and its thickness *t*_*m*_ ≈ 120 nm. The response is extracted from the transmission spectra of a MZI having one of its arms embedded with our non-Hermitian waveguide based thermo-optic phase shifter. For comparison, we also plot in the inset, a typical fall time of a conventional photonic phase shifter in silicon, with the heater placed well above the cladding (shown by dashed magenta line). (b) Measured electrical heating power required by our thermo-optic phase shifters to achieve a shift in resonance by one FSR (*π* phase shift). We estimate a *P*_*π*_ = 12.3 mW from the shift of the fringes (inset) with applied heating power.

Our non-Hermitian waveguide based thermo-optic phase shifters show an efficiency *P*_*π*_ ⋅ *τ* = 19.1 mW μs, five times lower than the state-of-art techniques [2]. The efficiency of thermo-optic efficiency of our phase shifters is estimated by independently measuring – (i) the response time (*τ*), and (ii) the electrical heating power (*P*_*π*_) required to spectrally shift the MZI response by half-free spectral range (FSR) (i.e. *π* shift). In order to measure the response time, we apply a transient electrical signal with a voltage swing of ±1 V to the integrated heaters of the non-Hermitian multi-mode waveguide and subsequently measure the temporal change in MZI transmission. Figure 5a shows the transient change in MZI transmission in response to electrical bias signal applied to the integrated heater for a non-Hermitian multi-mode waveguide of width *w*_*d*_ = 4 μm partially clad (*w*_*m*_ = 400 nm) with aluminum. For comparison, we also plot the measured fall time for a conventional multi-mode silicon waveguide but with the metal heater placed far away on top of the oxide cladding (inset) [24]. One can see that the response of the non-Hermitian waveguide-based thermo-optic phase shifter is over five times faster than that of the traditional heater placed over the waveguide cladding. We measure the response times of non-Hermitian waveguides with different widths (*w*_*d*_ and *w*_*m*_ ≈ *w*_*d*_/10). In Figure 5a-inset we present measured response times as a function of non-Hermitian waveguide width. One can see that unlike conventional heater design, our non-Hermitian waveguide based thermo-optic device shows a gradual decrease in response time with a reduction in waveguide width. We show in Figure 5b the electrical heating power required to induce a *π* phase shift in the transmission characteristics of the MZI (i.e. shift by half-FSR). We measure *P*_*π*_ = 12.3 mW for a device with non-Hermitian multi-mode waveguide length *L*_*D*_ = 150 μm, width *w*_*d*_ = 4 μm, metal width *w*_*m*_ = 400 nm, and *t*_*m*_ = 120 nm. In Figure 5b-inset, we show the evolution of electrical heating power with increase in waveguide width due to an increase in heat capacity. In this comparison, we have maintained a constant interaction length (*L*_*D*_ = 150 μm). We set the heater length to *L*_*D*_ = 50 μm and obtain propagation losses of 0.63 dB for the guided fundamental mode (in *w*_*d*_ = 4 μm waveguide), and an efficiency *P*_*π*_ ⋅ *τ* = 19.1 mW μs at the probe wavelength. In this case, the response time we measure as shown in the Figure 5a, is *τ*_*r*_ = 1.43 μs and *τ*_*f*_ = 1.44 μs. The response times are within the range of that found in literature; however, those works reported earlier showed much higher *P*_*π*_ [11], [22], [24]–[29].

**Figure 6:**
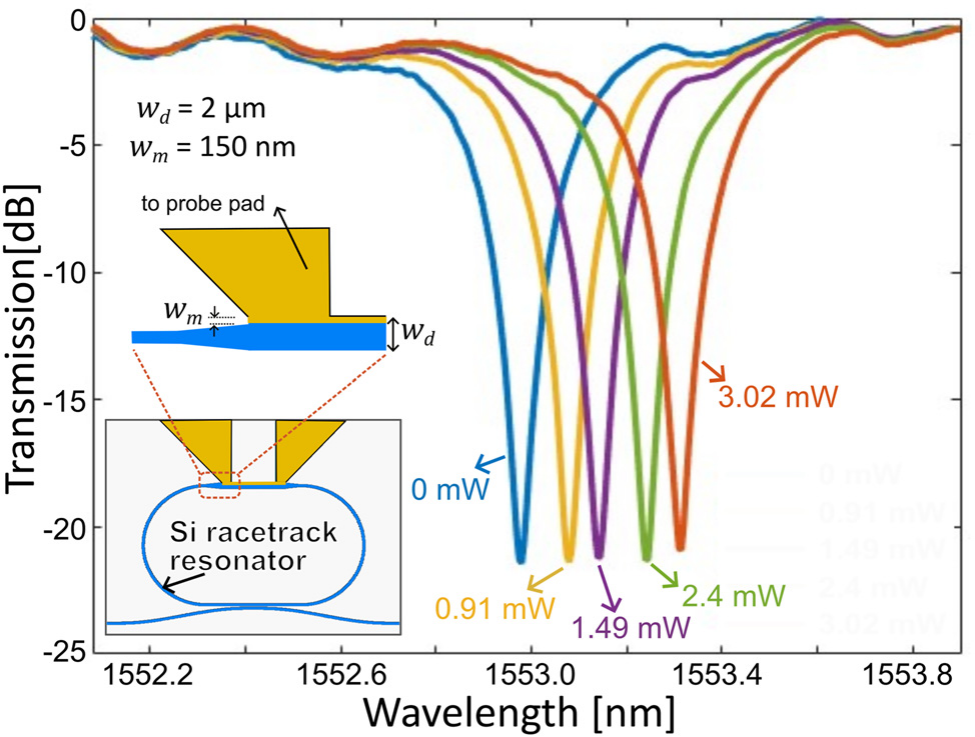
Integrated racetrack resonator embedded with non-Hermitian waveguides. Normalized transmission of the silicon racetrack resonator for different heater power. The resonator has an intrinsic quality factor of 10.3 × 10^3^. The resonance undergoes a shift by full-width half maxima for an electrical heating power of <1.5mW .

We further demonstrate the applicability of our non-Hermitian waveguide-based heater design by integrating it with a racetrack resonator. The racetrack resonator is prepared in an SOI platform with a standard single mode waveguide (*w* = 450 nm, *h* = 220 nm) but with one of the straight arms having a non-Hermitian waveguide with width *w*_*d*_ = 2 μm, length *L*_*D*_ = 20 μm, partial aluminum cladding width *w*_*m*_ = 150 nm, and its thickness *t*_*m*_ = 120 nm. In Supplementary Section 4, we present simulated mode loss for different width of metal coverage on a 2 μm wide silicon waveguide and find that our chosen metal cladding width here, is wide enough to introduce higher than threshold losses, ensuring operation above the loss-threshold point. Moreover, the shorter length of the non-Hermitian section (*L*_*D*_) here ensures lower losses and (provides) higher quality factors (*Q*). Figure 6 shows the measured transmission characteristics of our silicon racetrack resonator with a section of non-Hermitian waveguide embedded into it. We measure an intrinsic quality factor *Q*_0_ = 10^4^ and a resonance lifetime of 53 ps, appropriate for high bandwidth applications. The resonator requires a heating power of less than 1.5 mW for shifting the ring resonance by full-width half-maxima.

## 4 Conclusions

Using principles of a non-Hermitian system, we show that losses in photonic structures can be efficiently redistributed among different eigen-modes of the system [17], [30]–[33]. Furthermore, we employ this technique to demonstrate an efficient thermo-optic phase shifter using a metal-clad silicon waveguide. Instead of reducing loss by keeping lossy materials far away from the optical mode and subsequently having degraded performance, our work engineers the losses for improved performance. Our non-Hermitian waveguide based thermo-optic phase shifter presents a Figure of merit (*P*_*π*_ ⋅ *τ*) five times lower than state of the art thermal phase shifters achieved using complex strategies [24], [28], [34]–[39]. The improvement in thermo-optic response time (*τ*) in our waveguides does not come at the expense of power efficiency (*P*_*π*_) as observed in conventional heaters. Our demonstrated methodology opens up new avenues in integrated photonic-chip design, particularly towards realizing efficient active devices including modulators and lasers.

## Supplementary Material

Supplementary Material Details
