# Supplementary material for: Clearing a path for light through non-Hermitian media
Source: Nanophotonics. 2024 Aug 7;13(21):3945–52. doi: 10.1515/nanoph-2024-0140 (PMC11501056; doi:10.1515/nanoph-2024-0140)
Supplement: Supplementary file 1 — Supplementary Material Details [file j_nanoph-2024-0140_suppl_001.docx]

**Supplementary Section**

Clearing a path for light through non-Hermitian media

Utsav D. Dave, Gaurang R. Bhatt, Janderson R. Rodrigues, Ipshita Datta, and Michal Lipson

**1. Theory**

The general system of two waveguides, with mode amplitudes $a_{1}$and $a_{2}$, respectively having propagation constants *β*_1_ and *β*_2_, gain/loss *γ*_1_ and *γ*_2_ and with a coupling between them of *κ*_12_ is modeled as:

$i\frac{d}{dz}\left( \begin{matrix} a_{1} \\ a_{2} \end{matrix} \right)\mathcal{=H}\left( \begin{matrix} a_{1} \\ a_{2} \end{matrix} \right)$ (1)

$$\mathcal{H}\boldsymbol{=}\left( \begin{matrix} \beta_{1}-i\gamma_{1} & \kappa_{12} \\ \kappa_{12} & \beta_{2}-i\gamma_{2} \end{matrix} \right)$$

 (2)

The two eigenvalues of the system are:

$\beta_{\pm}=\beta_{avg}\pm\sqrt{\left| \kappa_{12} \right|^{2}+\left( \Delta\beta+i\Delta\gamma\right)^{2}}-i\gamma_{avg}$ (3)

Here, *β*_avg_ = (*β*_1_ + *β*_2_)/2 is the average propagation constant, Δ*β* = (*β*_1_ - *β*_2_)/2 is half the difference in propagation constants, *γ*_avg_ = (*γ*_1_ + *γ*_2_)/2 is the overall average system gain/loss, and Δ*γ* = (*γ*_1_ – *γ*_2_)/2 is half the gain/loss difference between the two waveguides. As can be seen from the above equation, the qualitative difference in the behavior of the two super-modes of the coupled waveguide system (corresponding to the two eigenvalues), is dictated by the term under the square-root sign.

The case of identical waveguides (in terms of dimensions and the real part of the index) with one of them being lossless and the other having loss, plotted in Fig. 1(a) of the main manuscript in solid lines, we have *β_1_* = *β_2_* = *β_0_*; and *γ*_1_ = 0 and *γ*_2_ > 0. In this case there is an exceptional point (EP) when *κ*_12_ = Δ*γ*. At this point, the two eigenvalues (and the eigenvectors) become identical and there is a non-Hermitian degeneracy, resulting in a reduction in the dimensionality of the system. The existence of the EP in the imbalanced gain/loss case can also be seen more clearly by rewriting the Hamiltonian as the sum of a perfectly *PT*-symmetric Hamiltonian *H*_PT_ (that hosts an EP) and an overall average system loss *γ*_avg_.

$\mathcal{H=}\mathcal{H}_{PT}+\mathcal{H}_{loss}$ (4)

where,

$\mathcal{H}_{PT}\boldsymbol{=}\left( \begin{matrix} \beta_{0}-i\Delta\gamma& \kappa_{12} \\ \kappa_{12} & \beta_{0}+i\Delta\gamma\end{matrix} \right);$ and $\mathcal{H}_{loss}\boldsymbol{=}\left( \begin{matrix} -i\gamma_{avg} & 0 \\ 0 & -i\gamma_{avg} \end{matrix} \right)$

Away from the EP, the two eigenvalues and eigenvectors regain their separate identities. In the PT-broken regime, i.e. when Δ*γ* > *κ*, the losses of the two super-modes bifurcate as shown in Fig. 1(a) of the main manuscript. One super-mode gets more loss as Δ*γ* increases while the other gets lower loss. When Δ*γ >> κ*, we get the loss of the two super-modes *α*_1,2_:

$\alpha_{1}=\gamma_{1}-\frac{\kappa^{2}}{2\Delta\gamma}; \alpha_{2}=\gamma_{2}+\frac{\kappa^{2}}{2\Delta\gamma}$ (5)

Clearly, in the limit, the loss for the two super-modes approaches loss of the individual waveguide loss. Thus, far away from the EP, the system has a low-loss and a high-loss super-mode. Physically, this is accompanied by the mode profiles of the two super-modes getting confined in the high-loss and low-loss waveguides as shown in Fig. 1(a) of the main manuscript.

For non-identical waveguides (i.e. asymmetric) case, plotted in Fig. 1(a) of the main manuscript in dotted lines, *β_1_* ≠ *β_2_* and *γ*_1_ = 0 and *γ*_2_ > 0, there is no longer an EP. Instead, there is an avoided crossing between the two eigenvalue curves. As shown in Fig. 1(a) of the main manuscript, for small values Δ*β* (Δ*β* = 0.15*κ* is shown in the dotted lines), and for Δ*γ >> κ*, the qualitative behavior of the loss of the two super-modes is pretty similar to the symmetric case even though there is no EP in this case. Thus, far away from the EP or avoided crossing region, when Δ*γ* is very large, we still see a low-loss and a high loss mode.


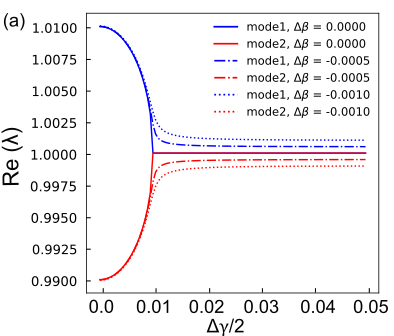

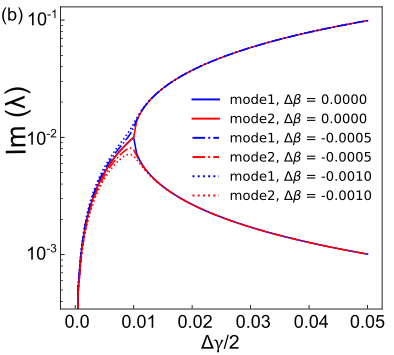


**Figure S1.** Computed real and imaginary part of the eigen value ($\lambda$) for two mode system with different $\Delta\beta$. The existence of the exceptional point with increase in loss in the system can be clearly seen for $\Delta\beta=0.$ With increase in $\Delta\beta$, one reaches an avoided crossing regime.

This behavior can in fact be generalized to *N* coupled waveguides. In general, such systems might possess a host of EPs of various orders if the parameters are tuned just right. Small deviations in those parameters will cause the avoided crossings to replace these EPs. However, we are interested in the behavior of the super-modes of the system far away from the EP/avoided crossing region since that is where we have the existence of low-loss super-modes (which can be verified numerically). When Δ*γ* is very large, these systems will in general host a series of low-loss and high-loss sets of super-modes. The system can be written as follows:

$i\frac{d}{dz}\left( \begin{matrix} a_{1} \\ \vdots\\ a_{N} \end{matrix} \right)\mathcal{=H}\left( \begin{matrix} a_{1} \\ \vdots\\ a_{N} \end{matrix} \right)$ (6)

$\mathcal{H}\boldsymbol{=}\left( \begin{matrix} \beta_{1}-i\gamma_{1} & \kappa_{12} & 0 & 0 & 0 & 0 \\ \kappa_{12} & \beta_{2}-i\gamma_{2} & \kappa_{23} & 0 & 0 & 0 \\ 0 & \kappa_{23} & \beta_{3}-i\gamma_{3} & \kappa_{34} & \cdots& \cdots\\ \cdots& \cdots& \cdots& \cdots& \cdots& \cdots\\ \cdots& \cdots& \cdots& \kappa_{N-2,N-1} & \beta_{N-1}-i\gamma_{N-1} & \kappa_{N-1,N} \\ 0 & 0 & 0 & 0 & \kappa_{N-1,N} & \beta_{N}-i\gamma_{N} \end{matrix} \right)$ (7)

This behavior is shown for a 6-mode system in Fig. 1(b) of the main manuscript where we can see a family of low-loss modes and a high-loss mode. As in the case of the two-mode system, the low-loss modes physically get confined to the low-loss region and the high-loss modes gets confined to the high-loss region. One can see this confinement from the simulated and experimentally measured mode profiles in Fig. 1(b) and Fig. 2(b) of the main manuscript respectively.

**2. Fabrication of non-Hermitian waveguides**

Non-Hermitian silicon waveguides are fabricated on standard silicon-on-insulator platform with device layer thickness of 220 nm. The patterns, including waveguide-based Mach Zehnder Interferometers (MZI), multimode sections, and race-track resonator-based structures are written using 100 KeV e-beam lithography on a negative-tone resist (ma-N 2403). The patterns are subsequently etched to create waveguide devices using an inductively coupled plasma etching system-employing SF_6_:C_4_F_8_ gasses in 1:1 ratio. The etch is performed at 8 mT chamber pressure with ICP Power = 2000 W and RF forward power = 20 W. Afterwards, the chips are thoroughly cleaned before another e-beam lithography step using positive tone resist (PMMA 950 A4) to define partially covering metal patterns on the waveguide. Partial metal cladding over the waveguide is achieved by sputter deposition of aluminum (Al) for a thickness of 120 nm followed by lift-off process. Thicker metal deposition ensured connection between partial metal clad on top of waveguide and metal via that run towards the contact pad in case of devices with heaters.

**3. Data analysis**

1. **Extracting loss from MZI data**

In order to extract loss due to non-Hermitian waveguide on the fundamental mode, we re-derive the MZI formulation for our case and fit the parameters to the measured spectral transmission shown in Figure 3. We assume MZI as the one shown in Figure S1. Let the time varying input light field, $E_{in}$, be applied to the input of the MZI. The field is split equally as $E_{1}$ and $E_{2}$, into two arms of the MZI via a multimode interference coupler (MMI) and gathered again at out MMI before the output field $E_{out}$. The output field can be written as:

$E_{out} =E_{1}+E_{2}$ (8)

Where the fields in the two arms in terms of input field amplitude ($A_{0}$) can be written as:

$E_{1}=\frac{A_{0}}{2}e^{-j\beta_{1}L_{1}}\cdot e^{-\alpha_{1}L_{1}/2}$

$$E_{2}=\frac{A_{0}}{2}e^{-j\beta_{2}L_{2}}\cdot e^{-\alpha_{2}L_{2}/2}$$

Here, $\beta_{1}$ and $\beta_{2}$, are the propagation constants of the fundamental mode in two arms of the MZI. The two arms have path lengths $L_{1}$ and $L_{2}$, and mode-field losses $\alpha_{1}/2$ and $\alpha_{2}/2$, respectively.


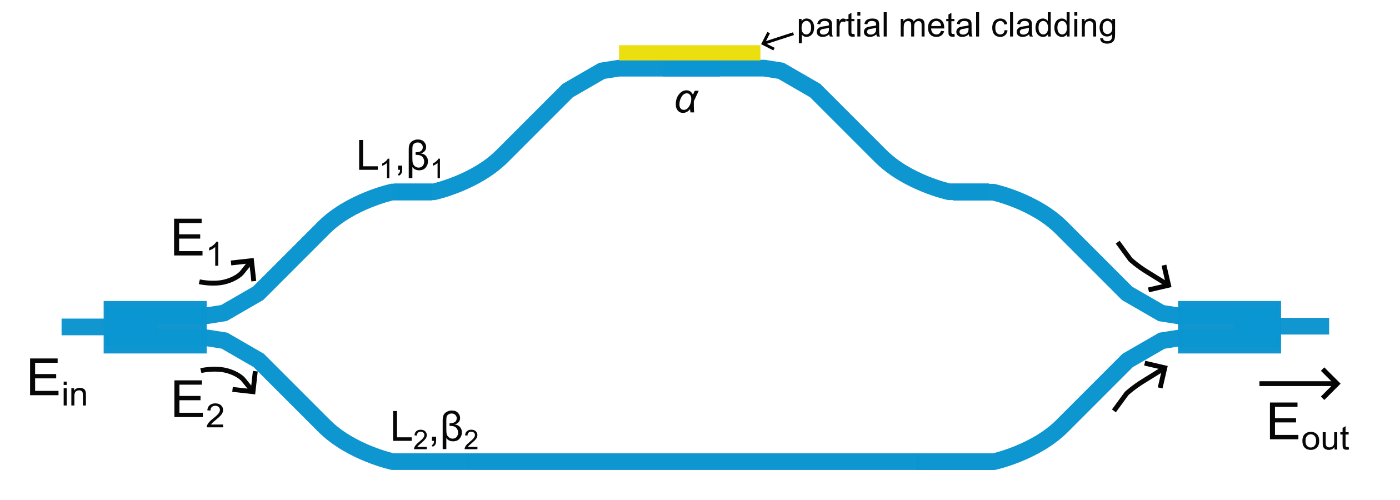


**Figure S2.** Schematic of an Mach-Zehnder Interferometer with one of the arm embedded with a non-Hermitian waveguide that introduces loss $\alpha$ in the fundamental mode and the second arm is a passive single-mode waveguide arm. The two arms of the MZI have a path length difference of $\Delta L=L_{1}-L_{2}$, where $L_{1}$ is the length of upper path with non-Hermitian waveguide and propagation constant $\beta_{1}$, while $L_{2}$ is the length of lower path having propagation constant $\beta_{2}$.

In order to accurately extract the loss from the partially metal-clad waveguide, its length is chosen to be long enough for its loss to be dominant and the length of the single-mode arm is chosen to be small enough such that its loss is negligible (i.e. $e^{{-\alpha}_{2}L_{2}/2}= 1$and, $e^{{-\alpha}_{1}L_{1}/2}= e^{-\alpha L_{1}/2}<1$). Subsequently, adding the field from two arms and solving for $E_{out}$, gives the following relation.

$E_{out}=\frac{A_{0}}{2}e^{-j\beta_{2}L_{2}}\left\{ {e^{-j(\beta_{1}L_{1}-\beta_{2}L_{2})}e}^{-\alpha L_{1}/2}+1 \right\}$ (9)

Solving for output power one gets the relation:

$P_{out}=\left| E_{out} \right|^{2}=p_{1}\cdot\left\{ 1+p_{2}+2\sqrt{p_{2}\cdot} cos(p_{3}) \right\}$ (10)

Where, $p_{1}=\left( \frac{A_{0}}{2} \right)^{2}$, $p_{2}=e^{-\alpha L_{1}}$, $p_{3}=\beta_{1}L_{1}-\beta_{2}L_{2}$

We fit the equation 9, to the measured data set to obtain the parameters $p_{1}$ , $p_{2}$ and $p_{3}$. Subsequently, from $p_{2}$, we obtain $\alpha$ using known design values for $L_{1}$.

1. **Extracting rise and fall time**

The time response measurement and data shown in Fig. 5(a) of the manuscript is fit to flowing equations.

For the rising edge,

$T = 1-e^{-t/\tau}$ (11)

For the falling edge,

$T=e^{-t/\tau}$ (12)


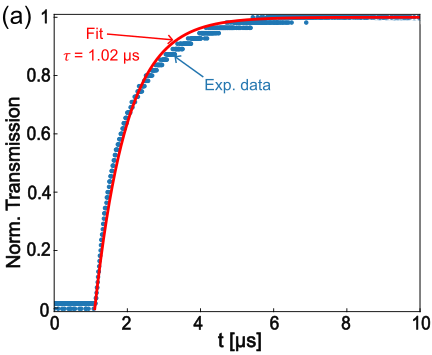

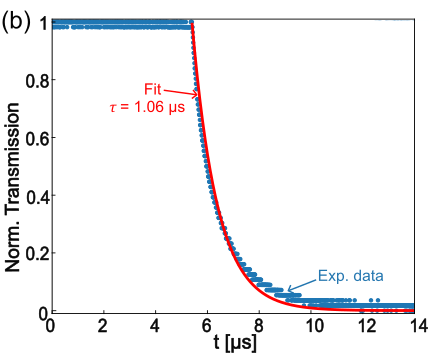


**Figure S3.** (a) Heater rising edge data and fit according to eq. 11. (b) heater falling edge and fit according to eq. 12.

1. **Loss in fundamental mode & first order mode with variation of metal coverage width**


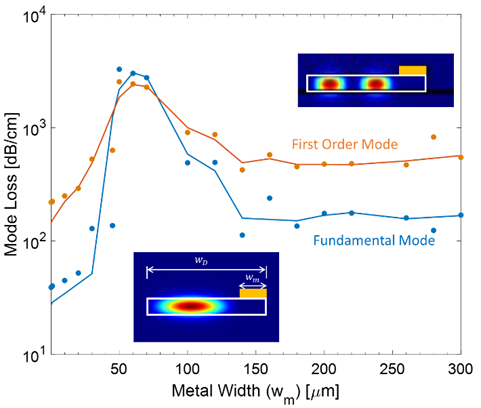


**Figure S4.** Computed mode loss of fundamental and first order mode for waveguide with width $w_{D}=2 \mu m$, and varying partial metal clad width ($w_{m}$). The fundamental mode loss increases initially with increase in metal clad width and reaches a maximum value before gradually reducing. The loss remains low for metal width higher than 150 nm. Ultimately the losses from metal dominate for all modes when the metal coverage is very high $(w_{m}>>600 \mathrm{nm}$, not shown here).
